# The effect of financial support on depression among young adults during the COVID-19 pandemic: Pierre-Julien Coulaud

**DOI:** 10.1093/eurpub/ckac129.746

**Published:** 2022-10-25

**Authors:** P Coulaud, T Salway, J Jesson, N Bolduc, O Ferlatte, K Bertrand, A Desgrées du Loû, E Jenkins, M Jauffret-Roustide, R Knight

**Affiliations:** 1 Department of Medicine, University of British Columbia, Vancouver, Canada; 2 BC Centre on Substance Use, Vancouver, Canada; 3 Faculty of Health Sciences, Simon Fraser University, Burnaby, Canada; 4 British Columbia Centre for Disease Control, Vancouver, Canada; 5 Centre for Gender and Sexual Health Equity, Vancouver, Canada; 6 School of Population and Public Health, University of British Columbia, Vancouver, Canada; 7 Centre d'Étude des Mouvements Sociaux, Paris, France; 8 Baldy Center on Law and Social Policy, Buffalo University, New York, USA; 9 School of Public Health, University of Montreal, Montreal, Canada; 10 Faculty of Medicine and Health Sciences, University of Sherbrooke, Montreal, Canada; 11 CEPED, Paris, France; 12 School of Nursing, University of British Columbia, Vancouver, Canada

## Abstract

**Background:**

To mitigate the adverse effects of the COVID-19 pandemic on financial resources, governments provided financial support (e.g., emergency aid funds) as well as family via personal assistance. This study aims to assess the moderating effect of financial support from the government or from family on the association between income loss and depression among young adults.

**Methods:**

Two online cross-sectional surveys among young adults (18-29) living in Canada and France were conducted in October-December 2020 (n = 4511) and July-December 2021 (n = 3329). Depressive symptoms were measured using PHQ-9 score+10. Two logistic regression models were performed for each survey with an interaction term between income loss and financial support (government or family modeled separately), controlling for demographics (e.g., country, age, gender, income, living conditions).

**Results:**

In the total sample, half reported depressive symptoms (2020/2021: 53%/46%), and over a third lost income (2020/2021: 10%/12% all income, 38%/22% some income). In 2020, 41% received government financial support (2021: 18%) while family/friends support was constant (12%). In both surveys, among those who received government support, income loss was associated with depression, whether participants lost all income (2020: AOR 1.75 [1.29-2.44]; 2021: AOR 2.17 [1.36-3.44]), or some income (2020: AOR 1.31 [1.17-1.81]; 2021: AOR 1.46 [0.99-2.16]). However, among those who received family support, income loss was no longer significantly associated with depression, whether participants lost all income (2020: AOR 1.37 [0.78-2.40]; 2021: AOR 1.51 [0.88-2.56]), or some income (2020: AOR 1.31 [0.86-1.99]; 2021: AOR 1.10 [0.67-1.81]).

**Conclusions:**

Association between income loss and depression was moderated by receipt of family financial support but not by receipt of government support. Financial support may help to mitigate the negative effects of income loss on young adults mental health during public health crisis.

**Key messages:**

• Financial support may help to minimize risk of depressive symptoms among youth who lost income related to the COVID-19 pandemic.

• Financial support through personal assistance (e.g., family, friends) appears to have a greater impact on youth mental health than COVID-specific government assistance funds.

